# Ecotin: A versatile protease inhibitor of bacteria and eukaryotes

**DOI:** 10.3389/fmicb.2023.1114690

**Published:** 2023-01-24

**Authors:** Frédéric De Meyer, Aurélien Carlier

**Affiliations:** ^1^Laboratory of Microbiology, Department of Biochemistry and Microbiology, Ghent University, Ghent, Belgium; ^2^LIPME, Université de Toulouse, INRAE, CNRS, Castanet-Tolosan, France

**Keywords:** protease inhibition, virulence, ecotin, host-associated bacteria, serpin

## Abstract

Serine protease inhibitors are a large family of proteins involved in important pathways and processes, such as inflammatory responses and blood clotting. Most are characterized by a precise mode of action, thereby targeting a narrow range of protease substrates. However, the serine-protease inhibitor ecotin is able to inhibit a broad range of serine proteases that display a wide range of specificities. This specificity is driven by special structural features which allow unique flexibility upon binding to targets. Although frequently observed in many human/animal-associated bacteria, ecotin homologs may also be found in plant-associated taxa and environmental species. The purpose of this review is to provide an update on the biological importance, role in host–microbe interactions, and evolutionary relationship between ecotin orthologs isolated from Eukaryotic and Prokaryotic species across the Tree of Life.

## Introduction

Serine-protease inhibitors (serpins) are a large family of protease inhibitors with members in bacteria, fungi, plants, and humans (Silverman et al., 2001; Spence et al., 2021). Serpins are primarily known for playing a role in controlling serine protease activity in biological processes (Gettins, 2002). Targets of serine proteases, in turn, participate in the regulation of a wide variety of complex physiological pathways, such as inflammation, fibrinolysis, and blood coagulation (Stein and Carrell, 1995). The structural mechanism by which serpins inhibit their protease substrates is well understood (Huntington et al., 2000). Target proteases interact with serpins, cleaving a reactive center loop (RCL) which protrudes from the serpin body. Following cleavage, but before hydrolysis of the acyl enzyme intermediate, the RCL inserts into the center of the serpin body, effectively trapping the protease. Through conformational changes, involving major reorganization of exposed protease recognition loops, serpins form a noncovalent complex upon binding (Gettins, 2000). Serpin protease inhibitors usually display high specificity (Gettins and Ofson, 2009), but some serpins are capable of inhibiting a broad range of serine proteases (Ksiazek et al., 2015).

**Figure 1:**
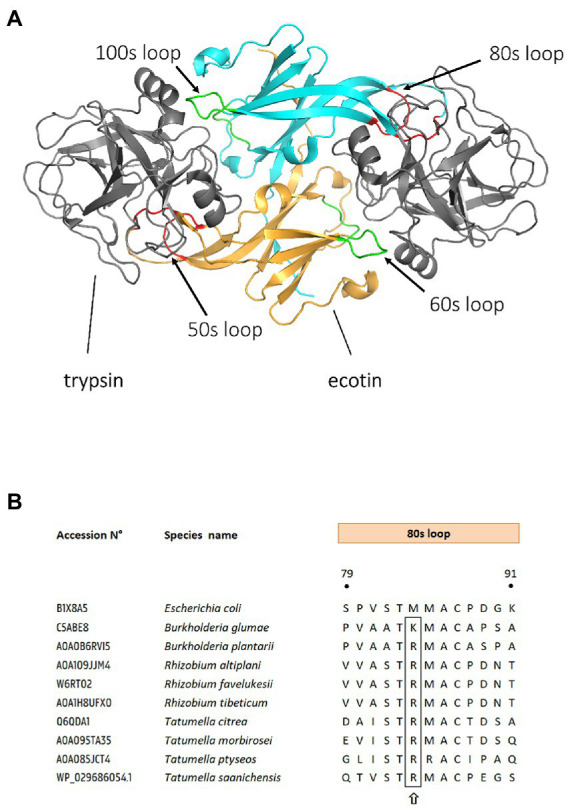
Binding sites of ecotin and ecotin-like proteins to their substrates. **(A)** Structure of a homodimeric ecotin in complex with trypsin protease. Ecotin (in orange and cyan) forms a stable homodimer, which binds two trypsin proteases simultaneously (in gray). The ecotin dimer contains two distinct protease-binding sites, each composed of two loops. The primary binding site (red) is composed of the 80s loop and the 50s loop, while the secondary binding site is composed of the 60s and 100 s loops (shown in green). Figure is adapted from Gillmor et al. (2000), using Protein Database (PDB) entry 1EZU. **(B)** Sequence alignment of ecotin orthologs primary binding site. Ecotin ortholog protein sequences were aligned, using MUSCLE (Edgar, 2004) in MEGA11 (Tamura et al., 2021). Residue position numbering is according to *E. coli* ecotin. The P1 position from the 80s loop (the primary specificity determinant) is designated by a upward arrow. UniProt or NCBI GenBank accession numbers are mentioned for each bacterial ecotin sequence.

Ecotin (*Escherichia coli* trypsin inhibitor) is a member of the serpin superfamily and a potent inhibitor of serine proteases, first isolated from *E. coli* (Chung et al., 1983). Its 16 kDa structure consists of a monomer that includes a 20 amino acid signal peptide which targets the protein to the periplasm (McGrath et al., 1991). High-resolution crystal structures revealed that two ecotin monomers assemble into a contralateral dimer which binds to two target protease molecules at opposite ends to form a heterotetramer (Pál et al., 1996; Yang et al., 1998). Each ecotin monomer inhibits its respective target *via* binding at two different surface contact sites: a primary and a secondary site (McGrath et al., 1994; Yang et al., 1998). This 1:1 stoichiometric configuration of two ecotin monomers for two protease units is unique among all the known structures and mechanisms of serine-protease inhibitor complexes (Figure 1A; Yang et al., 1998). Therefore, ecotin does not belong to one of the already established serpin families and has been classified in the MEROPS database as inhibitor family I11, clan IN (Rawlings et al., 2018). Interestingly, ecotin does not target endogenous *E. coli* proteases, and is therefore unlikely to be involved in the regulation of protease activity in this organism (Eggers et al., 2004). Rather, ecotin likely plays a role in protection against exogenous proteolytic attacks in environments such as the mammalian gastrointestinal tract (Chung et al., 1983; Seymour et al., 1994). In keeping with this hypothesis, ecotin is a reversible inhibitor of a wide range of mammalian and human derived serine proteases including trypsin, chymotrypsin, neutrophil elastase (NE), cathepsin G, granzyme B, and mannan-binding lectin-associated serine proteases (MASPs; Chung et al., 1983; Waugh et al., 2000; Nagy et al., 2019).

The structure and substrate binding properties of ecotin have been analyzed by X-ray crystallography and protease-binding assays (McGrath et al., 1994; Shin et al., 1996; Perona et al., 1997; Wang et al., 2001, 2003; Clark et al., 2011; Gaboriaud et al., 2013). These studies have primarily focused on the *E. coli* ecotin protein, but homologs from other taxa (e.g., *Yersinia pestis*) display similar protein quaternary structure and binding partners despite considerable primary sequence divergence (Clark et al., 2011). However, it is increasingly recognized that the genomes of diverse bacterial and eukaryotic taxa encode homologs of ecotin, which play various biological and functional roles (Eggers et al., 2004; Ireland et al., 2014; Verma et al., 2018; Nagy et al., 2019). This review aims at giving an update on the biological importance, role in host–microbe interactions, and evolutionary relationship between ecotin orthologs isolated from prokaryotic and eukaryotic species across the Tree of Life, as well as its potential in medical biotechnology.

## Taxonomic distribution of ecotin

**Figure 2:**
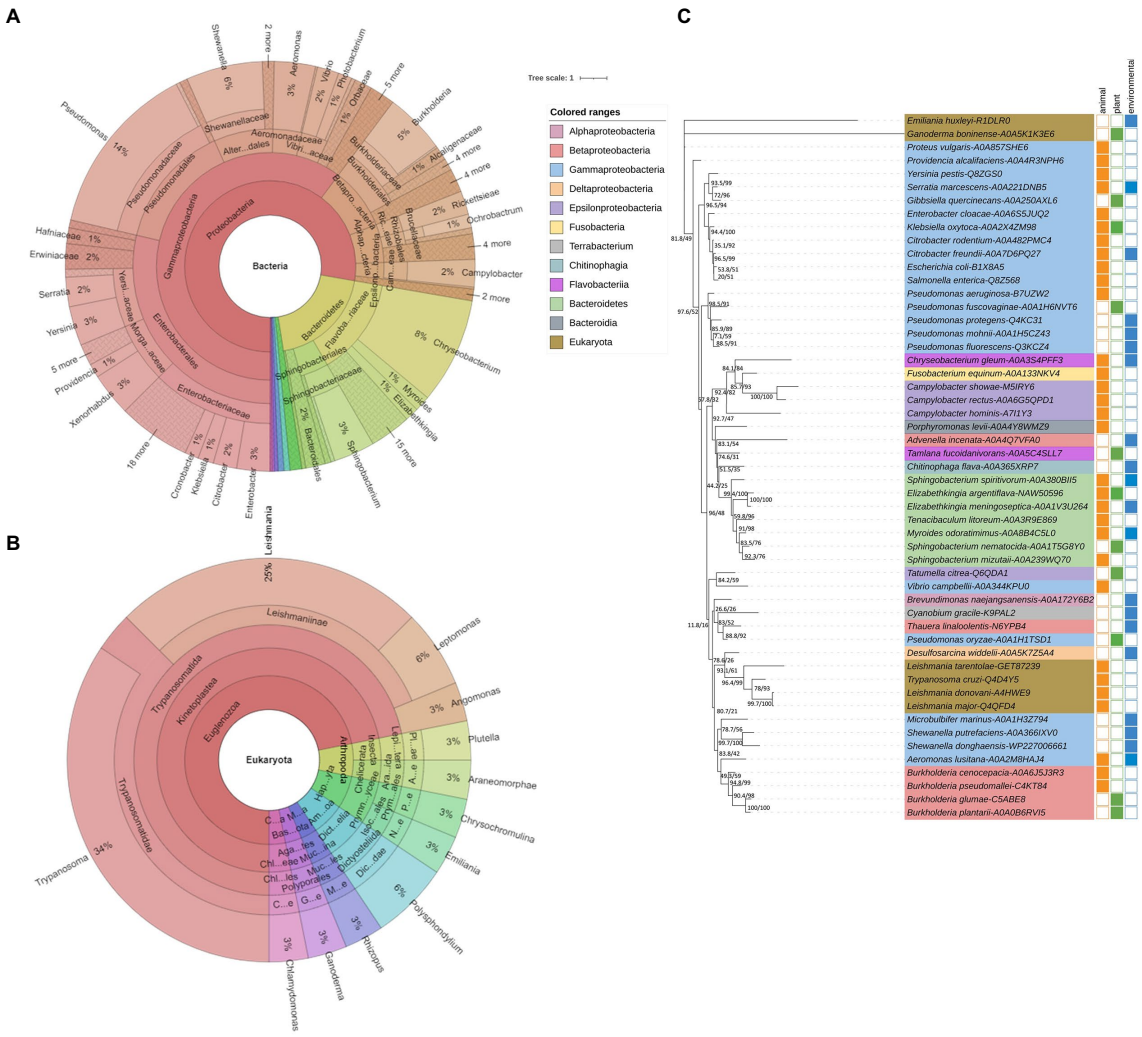
Taxonomic distribution of organisms containing ecotin orthologs. **(A)** More than six hundred species are present throughout the Tree of Life and encode at least one ecotin homolog. Protein sequences were extracted from InterPro (query: IPR036198) and validated as belonging to the same orthogroup with eggNOG-Mapper (E-value: 0.0001, minimum bit score: 60, % identity >40, query coverage >20%; Blum et al., 2021; Cantalapiedra et al., 2021). Orthology assignments were double checked in EggNOG database using EggNOG OGs. Remaining protein sequences were checked by the presence of ecotin domains as predicted by Pfam using eggNOG-Mapper (Mistry et al., 2021). One sequence for each species was retained, after which they were further aligned based on their amino acid sequence using MUSCLE (Edgar, 2004) in MEGA11 (Tamura et al., 2021). **(A)** Among *Bacteria*, the class of *Proteobacteria* (502 species) represents the majority of ecotin-expressing species. Note that this analysis is not comprehensive and homologs may have been missed due to biases in database representation and/or search parameters. **(B)** Among the Eukaryotes, both the *Trypanosoma* and *Leishmania* species, belonging to the *Kinetoplastida* class, contain over 10 species that are causative agents of several and widespread infectious diseases. The figure was made using KronaTools 2.7.1 (Ondov et al., 2011). **(C)** Unrooted phylogenetic tree of ecotin orthologs. Only one representative sequence per identified EggNOG OG was retained. Sequences were aligned based on their amino acid sequences using MUSCLE (Edgar, 2004) in MEGA11 (Tamura et al., 2021). A phylogenetic tree was constructed using the Maximum Likelihood (ML) method and LG + G4 substitution model in IQ-TREE (Minh et al., 2020). The SH-aLRT support (%) and the percentage of replicate trees in which the associated taxa cluster together in the bootstrap test (1,000 replicates) are shown next to the branches. The scale bar indicates the number of substitutions per site. Additional annotations are presented showing which taxa contain animal pathogenic (orange squares), plant-associated (green squares), and environmental (blue squares) strains.

Ecotin has been studied for nearly four decades (Chung et al., 1983), and more than 600 protein homologs have since been discovered across the Bacterial and Eukaryotic kingdoms (Figure 2; Eschenlauer et al., 2009; Ireland et al., 2014; Nagy et al., 2019; Garcia et al., 2020). Ecotin sequences diverge along the major taxonomic lineages, indicating that homologs were present in the ancestors of *Proteobacteria* (Figure 2C). Interestingly, sequences from other phyla (e.g., *Bacteroidetes*) are nested within *Proteobacteria* clusters, indicating that ecotin-like proteins were acquired *via* horizontal gene transfer (Figure 2C).

Ecotin homologs are widespread among Bacteria, particularly within the *Gammaproteobacteria* (Nagy et al., 2019). Among these, the genomes of several human pathogenic species such as *Yersinia pestis*, *Klebsiella oxytoca*, *Salmonella enterica, Citrobacter rodentium*, *Pseudomonas aeruginosa,* and *Burkholderia pseudomallei* and several species of *Enterobacter* encode ecotin homologs (Darby et al., 2014; Ireland et al., 2014; Tseng et al., 2018; Nagy et al., 2019; Salimiyan Rizi et al., 2019; Subramaniam et al., 2019; Vogt et al., 2019). Also in the *Gammaproteobacteria*, most genomes of *Xenorhabdus* species encode ecotin. Although this bacterial genus is known for symbiotic associations with nematodes, some members are also pathogenic to insects (Chaston et al., 2011). In addition, ecotin homologs have recently been characterized in pathogenic strains of *Campylobacter rectus* and *Campylobacter showae* (*Epsilonproteobacteria*), which reside in the oral cavity (Thomas et al., 2020).

Although mostly studied in the context of infection, ecotin proteins are not exclusive to animal pathogens. For instance, ecotin homologs are common in members of the genus *Pseudomonas*, which includes species without known pathogenic strains such as *Pseudomonas fluorescens* or *Pseudomonas protegens* (Figure 2A). *Pseudomonas* species are known for their metabolic diversity and ability to colonize a wide range of environmental niches (Spiers et al., 2000; Vartapetian et al., 2011), as well as the pathogenic *Pseudomonas aeruginosa*. Several other genera with few known pathogenic members encode ecotin homologs, including *Chryseobacterium* (more than 50 species) and *Shewanella* (more than 30 species). Although *Chryseobacterium* species have been isolated from diseased fish and human wounds, their main habitat is freshwater and soil (Gallego et al., 2006; Zhou et al., 2007; Cho et al., 2010). Similarly, *Shewanella* species are aquatic microorganisms with a worldwide distribution, with species adapted to extreme environments and are rarely host-associated (Abboud et al., 2005; Hau and Gralnick, 2007). Interestingly, ecotin homologs are also found in a few, taxonomically diverse plant-associated taxa. *Tamlana fucoidanivorans*, *Elizabethkingia argenteiflava,* and *Sphingobacterium nematocida* have been isolated from the endosphere or from plant surfaces (Liu et al., 2012; Li et al., 2020; Hwang et al., 2021). Furthermore, some of these species have a plant-pathogenic lifestyle. For example, *Pseudomonas fuscovaginae*, *Burkholderia glumae,* and *Burkholderia plantarii* are important pathogens of rice, while *Ganoderma boninense* and *Gibbsiella quercinecans* cause stem rot of oil palm and acute oak decline, respectively (Brady et al., 2016; Isha et al., 2020).

The genomes of several Eukaryotes also encode ecotin orthologs, especially within the phylum *Euglenozoa*, which contains the insect-borne parasitic genera *Trypanosoma* and *Leishmania* (Peña et al., 2017; Verma et al., 2017, 2018; Garcia et al., 2020; Levy et al., 2021). These genera contain species such as *Trypanosoma cruzi*, which causes Chagas disease (Brener, 1973), *Leishmania major* causing zoonotic cutaneous leishmaniasis (El-On et al., 1984), and *Leishmania donovani* the causative agent of visceral leishmaniasis, traditionally known as kala-azar (“black fever”; van Griensven and Diro, 2012). In contrast to prokaryotes which usually encode only 1 ortholog of ecotin, the eukaryotic unicellular *Leishmania* pathogens harbor three distinct paralogs, named ISP1, ISP2 and ISP3 (Eschenlauer et al., 2009; Morrison et al., 2012). In *L. major*, ISP1 is located on the same transcription unit upstream to ISP2 and ISP3 (Eschenlauer et al., 2009). ISP1 and ISP2 encode proteins of around 17 kDa, which is comparable to the 16 kDa mature form isolated from *E. coli* ecotin. ISP3, on the other hand, encodes a protein which is more than double the size (41.8 kDa), with an ecotin-like N-terminal domain, and a C-terminal domain of unknown function (Eschenlauer et al., 2009). Ecotin proteins of *Euglenozoa* form a distinct phylogenetic clade, nested within proteins of *Gamma*- and *Betaproteobacteria* (Figure 2C). Thus, ecotin proteins in these lineages seem to have evolved from a single ancestor, perhaps acquired from *Proteobacteria via* horizontal gene transfer. Interestingly, protease contact residues and substrate-like loops of *Leishmania* and *Trypanosoma* ecotin proteins display low identity to residues from bacterial homologs, indicating functional divergence (Peña et al., 2017).

## Function of ecotin in animal-associated microorganisms

Although the molecular structure and protease targets of ecotin are well characterized for a few organisms, its precise ecological function remains unclear. In *E. coli*, ecotin may play a role in protecting the cell against host proteases. Ecotin is translocated to the periplasmic space, where it can protect the cell against NE that may have permeated through the damaged outer cell membrane of Gram-negative bacteria (Eggers et al., 2004). Aside from a role in protection against the host immune system, ecotin from *E. coli* may play a role in microbe-microbe interactions, with *E. coli* ecotin knock-out strains more susceptible to T6SS-mediated killing by *Vibrio cholerae* (Myint et al., 2021). However, the precise mode of action remains to be elucidated. In *P. aeruginosa*, ecotin was shown to be released into the extracellular milieu *via* cell lysis during biofilm formation (Webb et al., 2003; Wang et al., 2013; Tseng et al., 2018). Ecotin homologs from *P*. *aeruginosa* and *Y*. *pestis* also inhibit NE (Eggers et al., 2001; Clark et al., 2011; Tseng et al., 2018). Similarly, ecotin of *B. pseudomallei* is essential for intracellular survival in murine macrophages, probably by inhibiting host proteases of the early endosome (Ireland et al., 2014). *P. aeruginosa* as well as *B. cepacia* are two major pathogens causing chronic infections in adult cystic fibrosis (*CF*) patients, both of which possess ecotin homologs (Govan and Deretic, 1996; Rajan and Saiman, 2002). Interestingly, *P. aeruginosa* and *B. cepacia* are opportunistic human pathogens that thrive in the lung environment as biofilms, and as such are not exposed to digestive or plasma proteases (Lavoie et al., 2011; Yaghi et al., 2020). However, pulmonary infections are mostly associated with increased numbers of degranulating neutrophils, and therefore by high concentrations of NE (Schaaf et al., 2000). A similar situation is also observed in *CF*, where there is an influx of neutrophils (Goldstein and Doring, 1986; Birrer et al., 1994; Witko-Sarsat et al., 1999). *P. aeruginosa* ecotin, released to the extracellular milieu *via* cell lysis during biofilm formation, directly binds to Psl, a component of the biofilm exopolysaccharide matrix (Tseng et al., 2018). Ecotin might therefore protect the biofilm from antimicrobial effectors and proteolytic degradation (Tseng et al., 2018; Nagy et al., 2019). This may represent a novel mechanism of protection for biofilms to increase their tolerance against the innate immune response. Interestingly, *P. aeruginosa* ecotin, together with the human protease inhibitor SERPINB1, has recently been proposed to act as a barrier to SARS-CoV-2 infection in *CF* lungs by inhibiting priming of the S protein by TMPRSS2 (Stanton et al., 2020). Although this role of ecotin as a protective layer may have intriguing consequences for the host, this remains to be proven experimentally.

The main function of the ecotin-like homologs ISP1, ISP2, and ISP3 of eukaryotic parasites *T. cruzi* and *L. major* is likely protection against intestinal proteases, such as neutrophil elastase in the gut of an insect vector (Lima and Mottram, 2010; Alam et al., 2016; Verma et al., 2017). However, a potential role for these proteins in survival inside the insect vector remains to be tested. Protozoan metacaspases (MCAs) of *Leishmania* species can be inhibited by the peptide ecotin-like ISP3 inhibitor from *L. amazonensis* and *L. major*. ISP3 interferes with the trypsin-like activity, resulting in significantly reduced parasite cell death (Peña et al., 2017; Shadab et al., 2017). In addition, serine proteases with MCA activity also play a role in the programmed cell death (PCD) in *Leishmania donovani* (Das et al., 2014), *Trypanosoma brucei* (T. brucei; Szallies et al., 2002), and other *Leishmania* species (Lee et al., 2007; Khademvatan et al., 2011; Castanys-Muñoz et al., 2012). Ecotin-like protein ISP2 of *L. major* inhibits mannan-binding lectin (MBL)-associated serine protease (MASP)-2 in addition to NE in the host cell (Verma et al., 2018). MASP-2 is involved in the cleavage of proteins in the complement system as well as in the coagulation cascade through cleavage of prothrombin to thrombin (Thiel et al., 1997; Krarup et al., 2007). In addition to *L. major*, *E*. *coli*, *Y*. *pestis,* and *P*. *aeruginosa* homologs also display inhibition of MASP2, but also of MASP1 and MASP3 with K_i_ values ranging from 10^−5^ to 10^−9^ (Cortesio and Jiang, 2006; Gaboriaud et al., 2013; Nagy et al., 2019). MASP1, MASP2, and MASP3 participate to the lectin pathway of the complement system, an essential part of the innate immune system that acts as the first line of defense against pathogens (Héja et al., 2012; Dobó et al., 2016). This function of ecotin seems surprisingly conserved, as endogenous ecotin also protects *E. coli* against attack from the lectin pathway (Nagy et al., 2019). Most recently, ISP2 from *T. brucei* has been identified as a virulence factor, contributing to the reduction of NO-producing myeloid cells and of IFN-γ-producing NK-cells: Mice infected with *ΔISP2* mutants strains displayed lower blood parasitemia, delayed symptoms, and survived longer (Levy et al., 2021). Moreover, ISP2 of *T. cruzi* contributes to evasion and replication in macrophages (Garcia et al., 2020). These findings indicate that ISP2 is a virulence factor in mice and attenuates the inflammatory response during early infection. One of the major roles of ecotin in animal pathogens may be to inactivate key proteases of the immune system.

## The role of ecotin in plant-associated bacteria

Although the role of ecotin in protecting pathogens or parasites against the mammalian innate immunity is relatively clear, there is a dearth of data about the targets of ecotin outside mammal systems. Some notorious plant pathogens encode ecotin homologs, for example *Burkholderia plantarii*, *Burkholderia glumae, Pseudomonas fuscovaginae,* or *Tatumella citrea* (*Ta. citrea*). So far, a single study by Eggers et al. analyzed the contribution of ecotin to virulence of the plant pathogen *Ta. citrea* (Eggers et al., 2001). Interestingly, ecotin from *Ta. citrea*, the phytopathogen responsible for pink disease in pineapples (Cha et al., 1997), displayed 1,000-fold weaker inhibition against human NE compared to proteins of *E. coli*, *Y. pestis,* or *P. aeruginosa* (Eggers et al., 2004). Despite this lower anti-NE activity, ecotin from *Ta. citrea* maintains inhibitory activity against trypsin, and may thus protect the bacteria against digestive proteases in an animal vector (Eggers et al., 2004). The first line of defense of plants is drastically different from the mammalian immune system. For example, NE is known to be involved in the non-oxidative pathway of innate defense which represents the first line of defense against invading microorganisms in mammals (Burg and Pillinger, 2001; Hargaden and Singer, 2012; Juul-Madsen et al., 2013), but there is no equivalent in plants (Thomas et al., 1988). Plants have developed various other mechanisms to protect themselves from pathogens. After the first contact with a pathogen, plant cells react by releasing reactive oxygen intermediates, salicylic acid, nitric oxide, ethylene, and/or jasmonic acid (Conrath et al., 2002). These signals orchestrate different downstream responses like the activation of cell wall reinforcement proteins or synthesis of antimicrobial peptides and phenolics, depending on the nature of the injury or infection (Jones and Dangl, 2006). Perhaps because of this divergence of targets, ecotin proteins in animal pathogens commonly display aliphatic methionine (M) or leucine (L) residues in the ecotin contact binding loop, whereas plant pathogens like *Ta. citrea* and *B. glumae* display conserved cationic arginine (R) and lysine (K) residues, respectively. Accordingly, ecotin of *Ta. citrea* only weakly inhibited mammalian thrombin, indicating the importance of other contact residues in the selectivity and binding specificity of the target (Eggers et al., 2004). Interestingly, an R residue is also present in the ecotin contact binding loop of all *Rhizobium* and some *Tatumella* species such as *Ta. morbirosei*, *Ta. ptyseos,* and *Ta. Saanichensis* (Figure 1B).

Serine proteases are abundant in plants, participating in numerous crucial processes such as plant immunity (reviewed in Figueiredo et al., 2018). Plant subtilases, a large family of plant serine proteases (MEROPS subfamily S8A), have a broad range of biological functions in plant development, but also in response to biotic and abiotic stresses (Schaller et al., 2012). Interestingly, some plant subtilases are key to the response to pathogen attack and PCD (Ryan and Pearce, 2003; Huffaker et al., 2006; Huffaker and Ryan, 2007; Vartapetian et al., 2011). As a countermeasure, some pathogens secrete inhibitors that target extracellular subtilases to avoid recognition. For example, tomato apoplastic S8 subtilases, P69B and P69C, are PR proteins that play a role in response to *Phytophthora infestans* and *Phytophthora syringae* infection (Jordá et al., 1999; Jorda et al., 2000). Kazal-like inhibitors EPI1 and EPI10 from *Phytophthora infestans* inactivate P69B, suggesting that protease inhibition is an important strategy for plant pathogens (Tian et al., 2004, 2005). Moreover, there are several other serine protease involved in different steps during plant immunity (Figueiredo et al., 2014; Van Der Hoorn & Klemenčič, 2021), but also in other processes during in plant defense activated through abiotic stimuli (Figueiredo et al., 2018). Metacaspases, for example, are also possible candidate targets as they are orthologs of metazoan caspases, restricted to fungi, protozoa and plants (Uren, 2000). Caspases are a family of cysteine proteases (C14), with a catalytic cysteine and histidine dyad essential for enzyme activity, playing a crucial role in PCD in plants (Del Pozo and Lam, 1998). Cysteine proteases have a distinct catalytic mechanism from serine proteases, and are important hubs in plant immunity (Misas-Villamil et al., 2016). Although there are no documented ecotin targets in plant immunity-related processes, the ecotin-like protein ISP3 from *L. amazonensis* has recently been suggested to also inhibit cysteine proteases such as MCAs (Peña et al., 2017). The presence of ecotin in the proteomes of plant pathogens is therefore intriguing, and could indicate a role in circumventing or preventing plant defenses. However, prediction of potential ecotin targets is difficult, and in addition several of the plant pathogens mentioned above are related to clinical pathogens that cause severe infections in humans. For instance, there are reports of *B. glumae* clinical infections (Weinberg et al., 2007), *Tatumella ptyseos* is known as a foodborne opportunistic pathogen and *Ta. saanichensis* has been isolated from a *CF* patient (Mardaneh et al., 2014; Tracz et al., 2015; Bourlond et al., 2019). Whether ecotin plays an adaptive role in plant pathogenicity or is rather a vestige from ancestral animal-associated lifestyles remains unanswered.

## Conclusion

Although ecotin homologs are widespread in the Bacterial and Eukaryotic kingdoms with diverse host-associated or environmental lifestyles, only a handful potential targets have been identified. Exploring and characterizing new ecotin targets could therefore be of importance in understanding several poorly studied pathways or protease reaction cascade mechanisms. Characterizing ecotin targets in new systems, especially plants, may provide novel insights into host immunity, and perhaps new ways to manage infections.

## Author contributions

FD and AC drafted and edited the manuscript. All authors contributed to the article and approved the submitted version.

## Funding

AC acknowledges support from the Flemish Fonds Wetenschappelijk Onderzoek under grant G017717N. AC also acknowledges support from the French National Research Agency under grant agreement ANR-19-TERC-0004-01 and from the French Laboratory of Excellence project “TULIP” (ANR-10-LABX-41; ANR-11-IDEX-0002-02). The funders had no role in study design, data collection and analysis, decision to publish, or preparation of the manuscript.

## Conflict of interest

The authors declare that the research was conducted in the absence of any commercial or financial relationships that could be construed as a potential conflict of interest.

## Publisher’s note

All claims expressed in this article are solely those of the authors and do not necessarily represent those of their affiliated organizations, or those of the publisher, the editors and the reviewers. Any product that may be evaluated in this article, or claim that may be made by its manufacturer, is not guaranteed or endorsed by the publisher.
